# Microindentation of fresh soft biological tissue: A rapid tissue sectioning and mounting protocol

**DOI:** 10.1371/journal.pone.0297618

**Published:** 2024-02-29

**Authors:** Clíona M. McCarthy, Kevin L. McKevitt, Sinéad A. Connolly, Isabel Andersson, Fiona C. Leahy, Siobhan Egan, Michael A. Moloney, Eamon G. Kavanagh, Colin Peirce, Eoghan M. Cunnane, Kieran D. McGourty, Michael T. Walsh, John J. E. Mulvihill

**Affiliations:** 1 Biomaterials Cluster, Bernal Institute, University of Limerick, Limerick, Ireland; 2 School of Engineering, University of Limerick, Limerick, Ireland; 3 Department of Vascular & Endovascular Surgery, University Hospital Limerick, Limerick, Ireland; 4 Department of Colorectal Surgery, University Hospital Limerick, Limerick, Ireland; 5 Health Research Institute, University of Limerick, Limerick, Ireland; 6 School of Chemical Sciences, University of Limerick, Limerick, Ireland; University of South Carolina, UNITED STATES

## Abstract

Microindentation of fresh biological tissues is necessary for the creation of 3D biomimetic models that accurately represent the native extracellular matrix microenvironment. However, tissue must first be precisely sectioned into slices. Challenges exist in the preparation of fresh tissue slices, as they can tear easily and must be processed rapidly in order to mitigate tissue degradation. In this study, we propose an optimised mounting condition for microindentation and demonstrate that embedding tissue in a mixture of 2.5% agarose and 1.5% gelatin is the most favourable method of tissue slice mounting for microindentation. This protocol allows for rapid processing of fresh biological tissue and is applicable to a variety of tissue types.

## Introduction

3D biomimetic models are powerful tools in better understanding disease processes. Data from experimental mechanical testing of fresh biological tissues are used to inform these 3D biomimetic models allowing researchers to better simulate *in vivo* conditions for a more robust understanding of the tissue extracellular matrix (ECM) [1–3]. While macroscale mechanical testing is useful for obtaining the bulk mechanical properties of biological tissue, this type of testing cannot capture the regional mechanical differences that occur in the ECM microenvironment [4–7]. Microindentation is a type of mechanical testing performed at the microscale and has the scope to detect these regional differences [7, 8]. However, detecting these regional differences requires that microindentation takes place within hours of tissue harvest to mitigate the effects of tissue degradation, thereby leading to a number of challenges associated with preparing the tissue for testing [8, 9]. The main challenges are the requirement that tissue sectioning and mounting occurs within a limited timeframe before the onset of tissue degradation [10, 11].

Additionally, to the authors’ knowledge, no standardised protocol exists for rapid sectioning and mounting of fresh biological tissue that can be applied to a variety of tissue types. The parameters used for fresh tissue sectioning and mounting of these tissue sections for mechanical testing vary widely in literature as shown in Table 1. The structural and compositional heterogeneity that exists within biological tissue samples adds an additional element of complexity to these tasks [12, 13]. For example, fresh biological tissue is difficult to section as it can tear easily, resulting in damage to the structure of the tissue [10, 14].

**Table 1:** Different tissue sectioning and sample mounting protocols used in literature.

Tissue	Embedding Reagent(s)	Section Thickness	Temperature	Timing	Mounting Reagent
Human Aortic Tissue	1 g Agarose in 50 mL 0.5× TAE buffer	150–700 μm	4°C	Not specified	Glued onto plastic tape [15, 16]
Human Umbilical Cord (Wharton’s Jelly)	N/A	1 mm		Not specified	Cell-Tak Transglutaminase [11]
Human Colon Tumour	2–4% Agarose	100 μm	On ice	6 hours 30 minutes (3 hours 30 minutes for embedding and sectioning, 3 hours for atomic force microscopy)	Poly-L-lysine-coated AFM-compatible glass bottom dish [10]
Human Liver and Intestine	3% Agarose	100–250 μm (Liver) 300–400 μm (intestine)	On ice	Up to 3 hours 30 minutes (not a microindentation study)	N/A [17]
Rat Liver and Intestine	3% Agarose	100–250 μm (Liver) 300–400 μm (intestine)	On ice	Up to 3 hours 30 minutes (not a microindentation study)	N/A [17]
Mouse Brain	2.5% Agarose	400 μm	4°C	Not specified	Histoacryl tissue glue [18]
Mouse Brain		300 μm		Atomic force microscopy is performed within 8 hours of preparation	0.05% polyethylenimine [9, 19]
Mouse Heart, Liver and Kidney	N/A	N/A	N/A	Nanoindentation testing within 6 hours of obtaining sample from surgery	Glue [7]
Porcine Colon	2.5% Agarose and 1.5% Gelatin	400 μm	On ice	Sectioning, mounting and microindentation within 2 hours	2.5% Agarose and 1.5% Gelatin [8]

The current gold standard for sectioning biological tissue is cryosectioning, but this procedure requires the tissue to be snap-frozen or fixed and snap-frozen beforehand. Freezing and fixing can lead to the alteration of some of the native mechanical properties of the tissue e.g. an increased elastic modulus when compared to fresh tissue [8]. Precision cut tissue slices (PCTS) are another method of preparing fresh biological tissue for studying the native microenvironment [17]. A vibratome is typically used to create PCTS, as it allows for tissue to be processed rapidly post-harvest from the host source, thereby maintaining the native tissue architecture [14, 17, 20, 21]. This method of tissue sectioning was first introduced in the 1980s and is widely used in research to prepare tissue slices for applications such as preparing tissue models, monitoring biochemical responses of tissues to pharmaceuticals and, more recently, microindentation of fresh biological tissue [8, 17, 20, 22]. PCTS for microindentation are required to be mounted and secured in place to create a stable testing environment ensuring that the native mechanical properties of the tissue are being captured.

The aim of this study is to develop and optimise a protocol for rapid tissue sectioning and mounting of fresh, soft biological tissue using a Compresstome® VF-210-0Z vibratome (Precisionary Instruments, Massachusetts USA), using fresh human femoral vein and porcine colon tissue. A vibratome is used in this study to enable this rapid fresh tissue sectioning and to mitigate the effect of tissue degradation. These slices are then used for downstream microindentation with the Optics 11 Chiaro Nanoindenter (Optics 11, the Netherlands). The microindentation data from these tissue samples can aid in the development of 3D biomimetic models, which better represent the ECM microenvironment.

## Materials & methods

The protocol in this peer-reviewed article is published on protocols.io DOI: 10.17504/protocols.io.q26g7pxm3gwz/v1 and is included for printing as a S1 File with this article.

A printable version of this protocol is available as S2 File with this article and A process flow on this protocol is available to download as S1 Fig for more information.

## Expected results

The criteria for a successful protocol are, firstly, a rapid tissue sectioning method so that tissue degradation is mitigated. Biological tissue has been shown to degrade between 4–24 hours post excision from the host source and mechanical testing should be performed immediately post tissue harvest to characterise representative native tissue properties [14, 20, 21, 23]. Table 1 shows the differences that exist in the timings of tissue sectioning and mounting between different studies ranging from 3 hours and 30 minutes (sectioning and mounting only) to 8 hours (including mechanical testing). The goal for this protocol is to have the tissue sectioned, mounted and microindentation performed in under 4 hours, before tissue degradation sets in. Additionally, it has been demonstrated that the stiffness of biological tissue increases when the tissue loses hydration [24–27]. It is therefore imperative that hydration is maintained during sectioning and mounting i.e. that the tissue is submerged in 1X phosphate buffered saline (PBS) for the duration of this protocol [24–27].

Another key component of a successful protocol is that the sectioned tissue needs to be mounted securely for downstream microindentation. As microindentation cannot be performed on unstable samples, the tissue samples should be secured in place so as not to float during testing [11]. Furthermore, the mounting reagent should be stiffer than the tissue tested, to avoid ambiguity around mechanical results obtained [28]. Various reagents such as glue, tape and 0.05% polyethylenimine are used for microindentation sample mounting with varying degrees of success [7, 9, 15, 16, 18] (Table 1).

The final criteria for a successful protocol is that it is applicable to a variety of tissue types. This not only provides for standardised testing across tissues, but also allows different tissues to be compared to each other both within and between studies, facilitating meta-analysis. The protocol developed in this study addresses these concerns by providing a standardised protocol for fresh tissue sectioning and mounting for microindentation.

## Results

### Time and hydration

Tissue sectioning, mounting and microindentation was performed in this study within 2 hours of receiving tissue to the laboratory. Equipment set up and reagent preparation was performed prior to receiving tissue. Both the human and porcine tissue were prepared for sectioning within 10 minutes. The embedding, sectioning and mounting of the tissue required approximately 40 minutes. The microindentation process required approximately 1 hour (as per the attached protocol). Tissue hydration was maintained throughout the protocol with 1X PBS apart from a single stage in the process where the tissue was mounted on to the specimen tube, as shown in the attached protocol.

### Mounting

Four typically employed mounting approaches were tested on both human femoral vein and porcine colon tissue to determine which was the most suitable for microindentation i.e. securing the tissue in place, number of successful indents (with low variability in the data) and clarity of sample under the microscope. The approach that satisfies these criteria would ensure that the data obtained is representative of the ECM microenvironment. The mounting conditions were as follows; glue (***G***), tape (***T***), glue and tape (***GT***), 2.5% agarose and 1.5% gelatin (***AG***). A sample of porcine colon tissue (n = 3 technical replicates per condition) and a sample of human femoral vein (n = 3 technical replicates per condition) were embedded in 2.5% agarose and 1.5% gelatin, as per the attached protocol, and sectioned into 400 μm sections. Once mounted, the samples were then used for microindentation.

**Fig 1:**
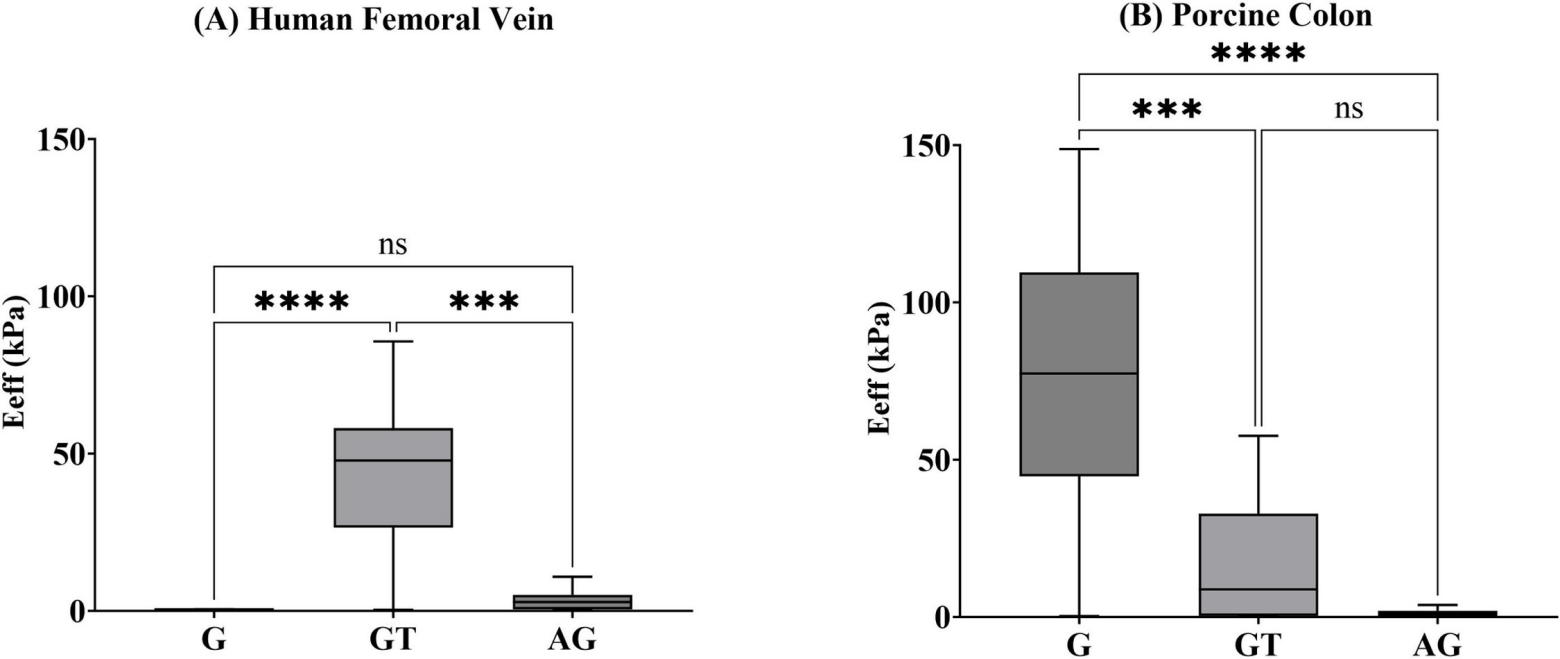
E_*eff*_ values for *G*, *GT* and *AG* in human femoral vein (A) showing a significant different between *G* and *GT* (p≤0.0001) and *GT* and *AG* (p≤0.001) for the human femoral vein. E_*eff*_ values for *G*, *GT* and *AG* porcine colon tissue (B) showing a significant different between *G* and *GT* (p≤0.001) and *G* and *AG* (p≤0.0001).

The raw microindentation data was analysed on the DataViewer Software V2.5.0 (Optics 11, the Netherlands) and is available as a supporting document S1 Dataset with this article. The effective elastic modulus (E_*eff*_) was calculated using loading curve of the Hertz contact model, with the depth limit of 16% of the probe tip radius applied i.e. 8 μm for a 50 μm probe tip radius. As a result, indents were excluded if the probe was in contact with the sample before the test began, if the probe did not find the surface of the sample or if the E_*eff*_ R^2^ value was < 0.9, as they were not considered successful indents. The results from the microindentation are shown in Table 2 and Fig 1 and the individual scales for each of the datasets are available as a S2 Fig with this article.

**Table 2:** Results for different mounting condition for downstream microindentation.

Tissue Type and Mounting Condition	Successful Indents (out of 27)	E_*eff*_ Values (kPa)
Human Femoral Vein ***G***	8	0.21 [0.11–0.45]
Human Femoral Vein ***GT***	20	47.80 [26.48–58.10]
Human Femoral Vein ***AG***	24	2.81 [0.49–5.05]
Porcine Colon ***G***	20	77.41 [44.74–109.6]
Porcine Colon ***GT***	17	8.79 [0.36–32.85]
Porcine Colon ***AG***	18	0.82 [0.25–1.96]

Statistical analysis was performed using Prism GraphPad Software V10.0.3 (GraphPad Software, La Jolla California USA). Outliers within the dataset were identified and removed if they exceeded 1.5 times the interquartile range below the 25^th^ percentile and above the 75^th^ percentile. The Shapiro-Wilk normality test was used to identify the most suitable testing requirements. The non-parametric Kruskal-Wallis ANOVA test was used to compare the E_*eff*_ for different mounting conditions for human femoral vein and porcine colon tissue respectively, as the data were not normally distributed. In this study, statistical significance was defined as a p value < 0.05. All data were presented in Fig 1 and Table 2 as median [25th– 75th percentiles] with Tukey boxplots.

Of the four different mounting approaches tested, only three were suitable for testing with microindentation. The samples mounted onto ***T*** did not adhere sufficiently to the surface and therefore began to float once 1X PBS was added. As a result, it was not possible to test these samples and this approach was rejected. The samples mounted onto ***G*, *GT*** and ***AG*** were sufficiently secured in place and microindentation was performed on the three remaining conditions using the method outlined in the attached protocol. While all three conditions had a degree of opacity when visualising the tissue regions of interest under the microscope, it was particularly difficult to visualise these regions with ***G*** and ***GT***.

Out of the 27 indents performed per mounting condition, the human femoral veins samples mounted onto ***G*** had eight successful indent E_*eff*_ values, the samples mounted using ***GT*** had 20 successful indent E_*eff*_ values and the samples mounted onto ***AG*** had 24 successful indent E_*eff*_ values. The E_*eff*_ values for the human femoral vein show a significant difference between ***G*** 0.21 [0.11–0.45] kPa and ***GT*** 47.80 [26.48–58.10] kPa (p≤0.0001) and between ***GT*** 47.80 [26.48–58.10] and ***AG*** 2.81 [0.49–5.05] (p≤0.001) (Fig 1(A)).

Out of the 27 indents performed per mounting condition, the porcine colon samples mounted onto ***G*** had 20 successful indent E_*eff*_ values, the samples mounted onto ***GT*** had 17 successful indent E_*eff*_ values and the samples mounted onto ***AG*** had 18 successful indent E_*eff*_ values. The E_*eff*_ values for the porcine colon show a significant difference between ***G*** 77.41 [44.74–109.6] kPa and ***GT*** 8.79 [0.36–32.85] kPa (p≤0.001) and between ***G*** 77.41 [44.74–109.6] kPa and ***AG*** 0.82 [0.25–1.96] (p≤0.0001) (Fig 1(B)).

### Versatility

As mentioned previously, the protocol is intended to be applicable to different tissue types. While protocols exist in literature for sectioning some tissues such as liver, intestine and brain tissue [10, 17, 18], there was a need for a standardised protocol that allows for rapid tissue sectioning and mounting of tissue post-harvest, that maintains tissue hydration throughout and is applicable to a range of different tissue types.

To demonstrate this, both human femoral vein and porcine colon tissue were tested in this study. The full list of biological tissues that this method has been applied to in our laboratory is shown in Table 3. It should be noted that 2.5% is the recommended agarose concentration for most soft biological tissues when using the Compresstome® VF-210-0Z [29] and the combination of agarose and gelatin allows the tissue to remain flat once sectioned [30]. However, for certain tissue types e.g. the human colon and porcine intestine the concentrations of embedding reagents may need to be doubled to provide sufficient stability for tissue sectioning [29]. Additionally the tissue slices used in this study were cut to 400 μm, as it allowed the native tissue architecture to be retained while also facilitating visualisation of the tissue regions of interest under the microscope. Other tissue types e.g. human colon (tumour) and porcine testes, remain intact at thicknesses of 250 μm and 200 μm respectively, thereby allowing for microindentation at these thicknesses.

**Table 3:** Tissue types and protocol parameters.

Tissue	Embedding Reagent(s)	Thickness Achieved	Temperature
**Human**			
Colon (Tumour)	Agarose 2.5% Gelatin 1.5%	250 μm	4°C
Colon (Healthy Margin)	Agarose 5% Gelatin 3%	300 μm	4°C
Veins (great saphenous, tibial, popliteal)	Agarose 2.5% Gelatin 1.5%	400 μm	4°C
Arteries (calcified ‐ femoral, popliteal)	Agarose 2.5% Gelatin 1.5%	400 μm	4°C
Carotid Plaque	Agarose 2.5% Gelatin 1.5%	400 μm	4°C
Abdominal Aortic Aneurysm	Agarose 2.5% Gelatin 1.5%	400 μm	4°C
**Porcine**			
Small Intestine	Agarose 2.5% Gelatin 1.5%	300 μm	4°C
Colon	Agarose 2.5% Gelatin 1.5%	300 μm	4°C
Liver	Agarose 2.5% Gelatin 1.5%	400 μm	4°C
Testes	Agarose 5% Gelatin 3%	200 μm	4°C

## Discussion

The objective of this study was to develop a method for rapidly sectioning and mounting fresh biological tissue prior to microindentation that is applicable to a number of biological tissue types.

### Time and hydration

Biological tissue has been shown to degrade as early as 4 hours post-harvest [14, 20, 23] and in this protocol, the total time for tissue embedding, mounting and mechanical testing was approximately 2 hours indicating the developed protocol was successful in achieving rapid tissue processing. Additionally, the effects of dehydration on tissue mechanical and structural properties have been demonstrated in literature [24–27, 31–33]. The protocol in this study aimed to maintain tissue hydration throughout the process to minimise the alterations that occur due to dehydration. Immediately post tissue harvest, the biological tissue samples were placed in 1X PBS and maintained in 1X PBS throughout the protocol. However there was a single stage in the protocol where this was not the case. During the embedding stage the tissue was removed from the 1X PBS and secured onto the specimen tube. This stage of the process lasts no longer than 5 minutes and, once embedded, the sample was placed back into 1X PBS for tissue sectioning. Post sectioning, the mounted tissue sample was submerged in 1X PBS for the duration of microindentation.

### Mounting

Sample mounting of soft biological tissues for mechanical testing is a challenging process due to the complexity of the tissue ECM, the tissue lifting during testing, or poor visualisation of the tissue regions of interest under the microscope due to reagent opacity [10, 11].

Four typically employed mounting approaches were compared for both human and porcine tissue to identify the method that is most suitable for microindentation. The condition ***T*** was rejected as the tissue was unable to adhere to the surface of the tape. The mounting condition using ***G*** was effective in securing the tissue in place, but when dry, the glue was opaque in colour. The presence of opaque glue makes the regions of interest of the tissue difficult to distinguish using a brightfield microscope. The visualisation of these tissue regions with the ***GT*** mounting condition once again proved difficult due to the opacity of the glue when dry. Although the ***AG*** had some opacity upon gel formation, it was still possible to view the regions of interest under the brightfield microscope.

**Fig 2:**
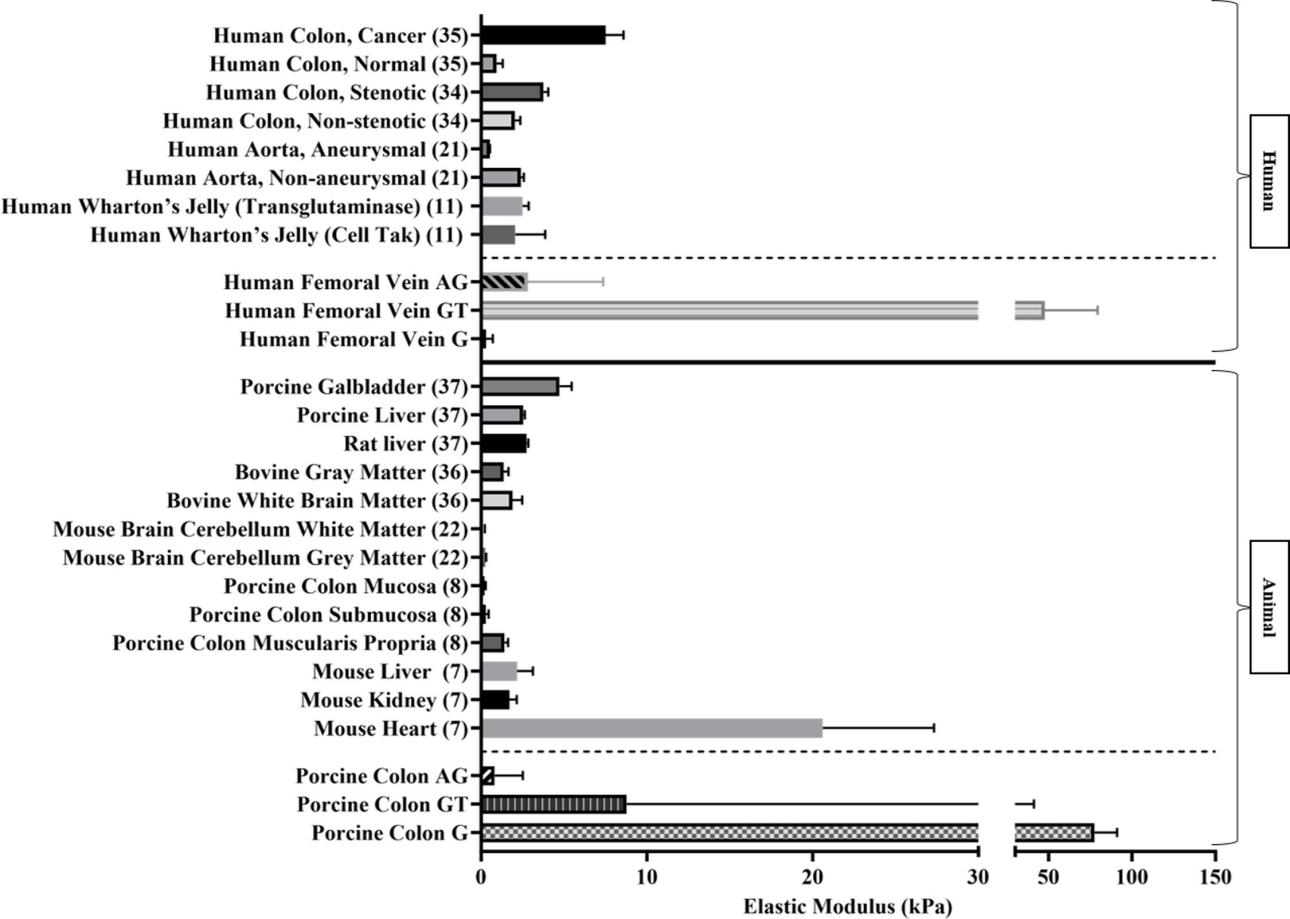
Elastic modulus values from literature compared to the elastic modulus values from this study [7, 8, 11, 16, 18, 34–37].

The mounting condition ***G*** exhibited the highest variability of successful overall indents compared to the remaining conditions tested, achieving eight indents for human femoral vein and 20 indents for porcine colon out of a maximum of 27 indents. While the data for human femoral vein has a low interquartile range in E_*eff*_ values (0.21 [0.11–0.45] kPa) the inverse was the case for porcine colon with a large interquartile range in E_*eff*_ values (77.41 [44.74–109.6] kPa) (Table 2). This may suggest that insufficient usable data were obtained with this mounting approach for the human femoral vein to give an accurate representation of the tissue biomechanics. Gluing tissue in place has also been linked to the introduction of artefacts, which can distort the mechanics of the tissue [32]. This appears to be the case with ***G*** for porcine colon and with ***GT*** for both tissue types. While ***GT*** had more successful overall indents than ***G*,** with 20 successful indents for human femoral vein and 17 successful indents for porcine colon, the data for this condition had the largest interquartile range in E_*eff*_ values of all mounting conditions i.e. 47.80 [26.48–58.10] kPa for human femoral vein and 8.79 [0.36–32.85] kPa for porcine colon (Table 2). The mounting condition ***AG*** had the highest number of successful overall indents of the tested mounting conditions with 24 indents for human femoral vein and 18 indents for porcine colon. Further, the E_*eff*_ values for human femoral vein (2.81 [0.49–5.05] kPa) and porcine colon (0.82 [0.25–1.96] kPa) had the lowest interquartile range of the mounting conditions, demonstrating the repeatability and low variability of this condition (Table 2). It was worth noting that biological tissue is heterogeneous and the elastic modulus values can vary between samples, as shown in the data from literature compared to the results from this study in Fig 2. However, the interquartile ranges in E_*eff*_ values for the human femoral vein for ***GT*** and the porcine colon for ***G*** exceed beyond this variability. ***AG*** was deemed the most suitable mounting condition as it sufficiently secured the samples in place, there was sufficient visibility of the samples under the microscope and it produced the most successful indents with the lowest variability of data.

### Versatility

The final objective was to demonstrate the application of the newly developed protocol to a number of different tissue types. Examples of these tissue types are shown in Table 3 for both human and porcine tissues. However, this protocol was not without its limitations. Due to the heterogeneity of biological tissue, minor adjustments were made to the protocol parameters for some tissues to prevent the tissue tearing. For example, while the embedding reagent concentration of 2.5% agarose and 1.5% gelatin was applied to most tissue types in this study. Some tissues proved more difficult to section and required the concentrations of the embedding reagents to be doubled (human colon healthy margins and porcine testes), as the embedding reagents should be approximately the same density as the tissue [29]. Certain parameters such as embedding reagent concentration and the vibratome approach speed and oscillation frequency were not investigated in this study and future work could be done on optimising this process.

## Summary

This study aims to develop and optimise a protocol for rapid tissue sectioning of fresh soft biological tissue using a Compresstome® VF-210-0Z vibratome (Precisionary Instruments, Massachusetts USA), which can be used for downstream microindentation with the Optics 11 Chiaro Nanoindenter (Optics 11, the Netherlands). The method outlined in this study allows for rapid sectioning of fresh biological tissue (while maintaining hydration), using a suitable mounting reagent and can be applied to a range of soft biological tissues. The data gathered can inform 3D biomimetic models that are more representative of the native ECM microenvironment. Our lab has a publication on the successful application of this method on porcine colon tissue with further publications under development [8].

## Supporting information

S1 File Step-by-step protocol, also available on protocols.io. (PDF)

S2 File Step-by-step protocol. Printable version for lab use.
(DOCX)

S1 Fig Process flow on rapid sectioning and mounting protocol. (TIF)

S2 Fig Figure on individual scales for microindentation data. (TIF)

S1 Dataset Raw and processed mechanical data for human femoral vein and porcine colon, including descriptive statistics. (XLSX)
