# Supplementary material for: Microindentation of fresh soft biological tissue: A rapid tissue sectioning and mounting protocol
Source: PLoS One. 2024 Feb 29;19(2):e0297618. doi: 10.1371/journal.pone.0297618 (PMC10903917; doi:10.1371/journal.pone.0297618)
Supplement: S1 File — (PDF) [file pone.0297618.s001.pdf]

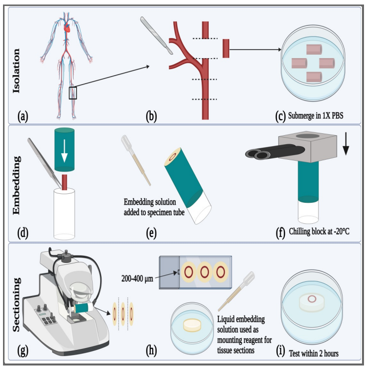

Microindentation of Fresh Soft Biological Tissue: A Rapid Tissue Sectioning and Mounting Protocol

Clíona M. McCarthy[1,2], Kevin L. McKevitt[3], Sinéad A. Connolly[1,2], Isabel Andersson[1,2], Fiona C. Leahy[3], Michael A. Moloney[3], Eamon G. Kavanagh[3], Eoghan M. Cunnane[1,2,4], Kieran D. McGourty[1,2,5], Michael T. Walsh[1,2,4], John J.E. Mulvihill[1,2,4]

[1]Biomaterials Cluster, Bernal Institute, University of Limerick, Limerick, Ireland;
[2]School of Engineering, University of Limerick, Limerick, Ireland;
[3]Department of Vascular & Endovascular Surgery, University Hospital Limerick, Limerick, Ireland;
[4]Health Research Institute, University of Limerick, Ireland;
[5]School of Chemical Sciences, University of Limerick, Ireland

Sinéad A. Connolly: Current address Laboratory of Biosensors and Bioelectronics, Institute for Biomedical Engineering, ETH Zürich, Zürich, Switzerland;

John J.E. Mulvihill

Protocol Info: Clíona M. McCarthy, Kevin L. McKevitt, Sinéad A. Connolly, Isabel Andersson, Fiona C. Leahy, Michael A. Moloney, Eamon G. Kavanagh, Eoghan M. Cunnane, Kieran D. McGourty, Michael T. Walsh, John J.E. Mulvihill . Microindentation of Fresh Soft Biological Tissue: A Rapid Tissue Sectioning and Mounting Protocol. **protocols.io** https://protocols.io/view/microindentation-of-fresh-soft-biological-tissue-a-c23jygkn

Created: Oct 06, 2023

Last Modified: Oct 13, 2023

PROTOCOL integer ID: 88907

Keywords: Tissue preparation, Vibratome, Fresh tissue slices, Precision cut tissue slices, Microscale testing

Funders Acknowledgement:
Irish Research Council Enterprise Partnership Scheme with Becton Dickinson
Grant ID: EPSPG/2020/467

ABSTRACT

Microindentation of fresh biological tissues is necessary for the creation of 3D biomimetic models that accurately represent the native extracellular matrix microenvironment. However, tissue must first be precisely sectioned into slices. Challenges exist in the preparation of fresh tissue slices, as they can tear easily and must be processed rapidly in order to mitigate tissue degradation. In this study, we propose an optimised mounting condition for microindentation and demonstrate that embedding tissue in a mixture of 2.5% agarose and 1.5% gelatin is the most favourable method of tissue slice mounting for microindentation. This protocol allows for rapid processing of fresh biological tissue and is applicable to a variety of tissue types.

IMAGE ATTRIBUTION

Images created with BioRender.com where cited. License number UF25YG57EN

GUIDELINES

Process the tissue immediately on arrival to the laboratory.

Maintain tissue hydration in 1X PBS throughout the experiment.

MATERIALS

pH Meter

50 mL Glass Bottle

Microwave (800W)

Water bath between 34-37 °C

Pasteur pipette

Compresstome® VF-210-0Z (Precisionary Instruments, Massachusetts USA)

Chilling block (VF-VM-CB-20-BOS) (-20 °C) (Figure 1a)

Buffer tray (VF-BT-VM-LP-BOS) (Figure 1b)

Specimen tube - white plunger (VF-SPS-VM-20-BOS) (Figure 1c)

Specimen tube - metal tube (VF-SPS-VM-20-BOS) (Figure 1d)

Acetone bath for blade holder (VF-VM-AB) (Figure 1e)

Allen wrench (VF-BH-VM-AW) (Figure 1f)

Blade holder (VF-VH-VM-210-0Z-BOS) (Figure 1g)

Stainless steel double-edged razor blades

Chiaro Nanoindenter (Optics 11, the Netherlands)

Scissors

Human tissue/porcine tissue

Scalpel (disposable)

Tweezers

Plastic cutting board

Blotting paper

Crushed ice

Paintbrush (5 mm flathead)

Superglue (Gorilla Superglue Micro Precise 5g)

60 mm Petri dishes

Glass slide (26x76 mm 1.0-1.2 mm)

1X Phosphate buffered saline (PBS) (chilled at between 0-4 °C)

Agarose MP (REF 11388983001)

Gelatin (from bovine skin G9391-100G)

Acetone (ACS reagent ≥99.5% PCode 102439046)

Note

Other brands for consumables are available

SAFETY WARNINGS

Wear appropriate PPE for the duration of the experiment i.e. lab coat, disposable gloves and safety glasses).

Refer to the appropriate safety data sheet for the reagents and chemicals for correct handling and storage.

Dispose of all sharps in a dedicated sharps bin.

Dispose of used reagents in the appropriate waste bins.

ETHICS STATEMENT

The study is approved by the institutional review board and is conducted in accordance with ethical guidelines (Research
Ethics Committee #118/18 and 100/15 (vascular tissue) and #031/2021 (colorectal tissue). Patient recruitment is carried out with informed consent.

BEFORE START INSTRUCTIONS

Equipment set up and reagent preparation is performed prior to receiving tissue into the laboratory.

Preparation of Phosphate Buffered Saline (PBS)

1 Phosphate Buffered Saline (PBS) is used as a buffer for tissue sectioning and as a solvent for the agarose and gelatin embedding solution. Prepare 1 L of a 10X concentration of PBS stock solution by dissolving 80 g NaCl, 2 g KCl, 17.8 g Na_2HPO_4 and 2.7 g KH_2PO_4 into 1 L of deionised water. The pH of the PBS is set at 7.4 with a pH meter by adding 5M NaOH to the solution. For the experiment, prepare 1 L of 1X PBS by adding 900 mL of deionised water to 100 mL 10X PBS. The solution is kept at 0-4 °C on melting ice. 1X PBS is also used as a solvent for the embedding solution.

Preparation of 2.5% (wt/vol) agarose and 1.5% (wt/vol) gelati...

2 30m

Weigh 1 g agarose and 0.6 g gelatin in a 50 mL glass bottle and add 40 mL of PBS to make a 2.5% concentration of agarose and 1.5% gelatin solution. According to the Compresstome® VF-210-0Z (Precisionary Instruments, Massachusetts USA)manual 2.5% agarose is the optimum concentration for cutting soft biological tissues such as cardiac, kidney and intestinal

tissue. Heat the bottle in the microwave until the agarose and gelatin have dissolved, stopping the microwave
every 10-15 seconds to mix the solution with a spatula spoon to ensure the agarose and gelatin are fully dissolved. Once ready, place the bottle in a water bath between 34-37 °C until required for embedding. Do not leave the embedding solution for longer than 00:30:00, as the embedding solution will solidify after this time point.

Note

Concentration of embedding solution may need to be doubled for certain tissue types such as human intestine and porcine testes which have higher amounts of collagen content.

Equipment Setup: Vibratome

3 The Compresstome® VF-210-0Z (Precisionary Instruments, Massachusetts USA) is set up according to the manufacturing guidelines and is shown in Figure 2a. Briefly, this vibratome uses controllable oscillations and speed to slice through tissue without the use of freezing or fixation of the fresh biological tissue. The oscillation frequency and speed settings are 14 Hz and 14 mm/s respectively. The razor blade is prepared with scissors as per the user manual and attached to the blade holder using superglue (Figure 1g). The chilled 1X PBS is added to the buffer tray just before sectioning commences (Figure 1b).

4

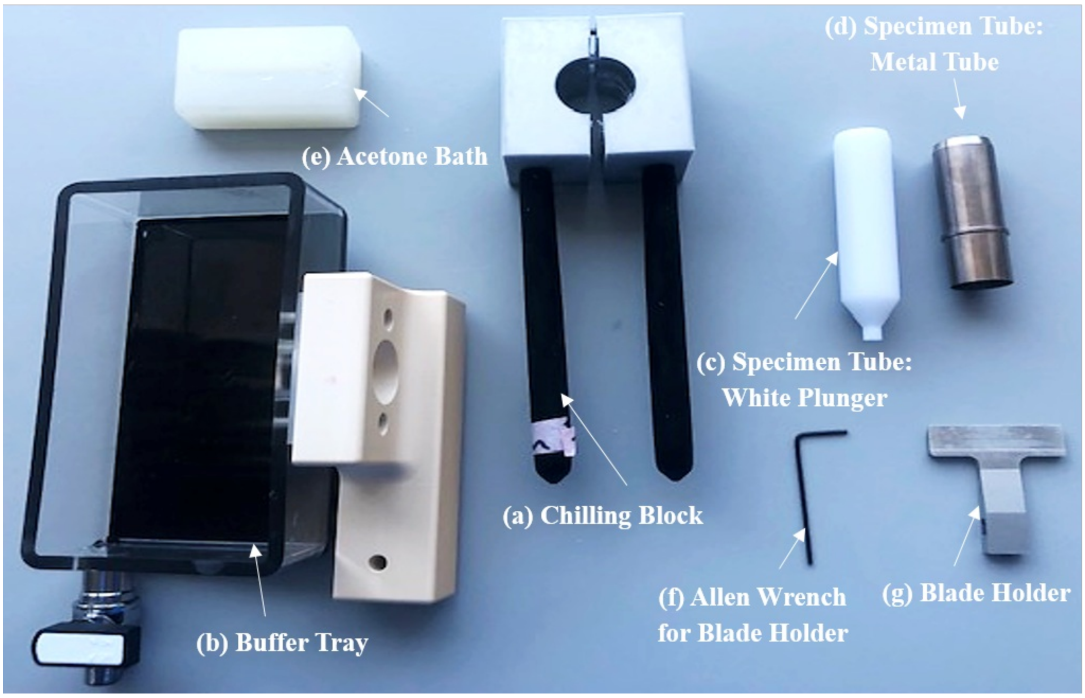

Figure 1 Compresstome® VF-210-0Z model components (1)

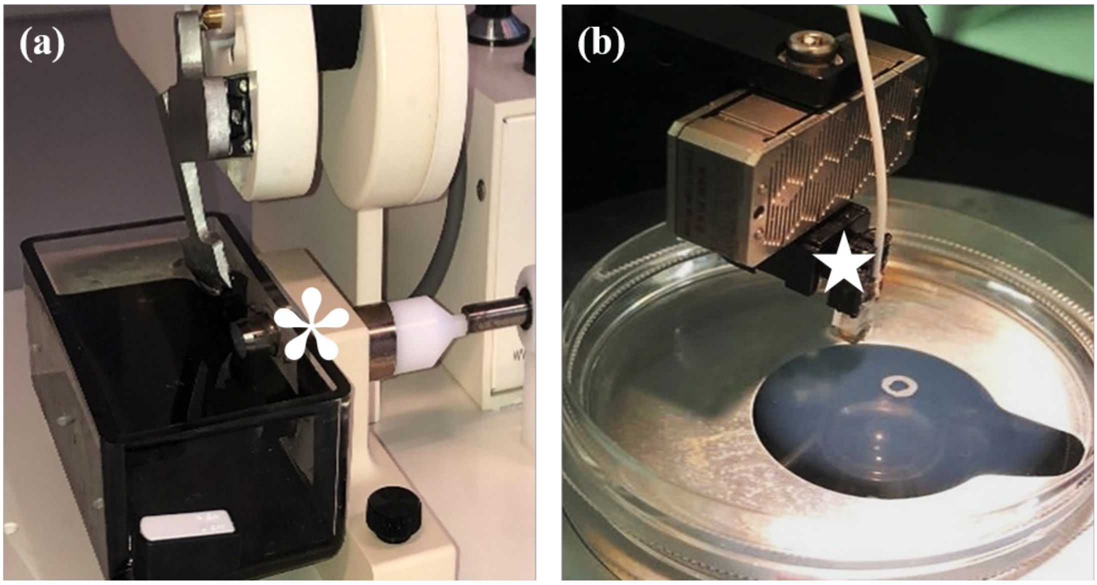

Figure 2 Setup of the equipment used in this study i.e., the Compresstome®VF-210-0Z and the Chiaro Nanoindenter. (a) The Compresstome® VF-210-0Z buffer tray is secured in place and the embedded specimen (white asterisk) is awaiting sectioning. (b) The Chiaro Nanoindenter has a calibrated probe (white star) mounted and ready for performing microindentation on a hydrated vascular sample.

Preparation of fresh human tissue for sectioning with vibrato...

10m

4 Human vascular tissue is sourced from the Department of Surgery, University Hospital Limerick, Limerick, Ireland. The study is approved by the institutional review board and conducted in accordance with ethical guidelines (Research Ethics Committee #118/18 and 100/15 (vascular tissue) and #031/2021 (colorectal tissue). Patient recruitment is carried out with informed consent. Segments of tissue specimens for testing are selected by the operating surgeon and placed into

0-4 °C 1X PBS immediately post-surgical excision from the patient as this mitigates tissue degradation (4, 5). The tissue specimens are transferred into 1X PBS and transported to the University of Limerick in an insulated cooler box, to maintain a temperature of approximately 0-4 °C The tissue is cut with the scalpel into 10 mm^2 sections and placed onto a petri dish. The tissue sections are submerged in 1X PBS and placed on melting ice between 0-4 °C to cool for approximately 00:05:00 , Figure 3a-c.

Preparation of porcine human tissue for sectioning with vibra...

10m

5 Porcine tissue is sourced from a local abattoir (Ballylanders, Co. Limerick, Ireland), which allows for testing within 2-3 hours of sacrifice (animals are already sacrificed for consumption and not for the purpose of this experiment). It is then placed into 0-4 °C 1X PBS immediately post harvest, as this promotes tissue viability (4,5). From the tissue samples, using the scalpel, an approximately 40 mm length of tissue is cut and excess fat is removed from this section. Next, the section is cut longitudinally and washed multiple times in 1X PBS to remove mucous, bile and excrement etc. The tissue is cut into 10 mm^2 sections and placed onto a petri dish. The tissue sections are submerged in 1X PBS and placed on melting ice between 0-4 °C to cool for approximately 00:05:00 .

Procedure for the fresh biological tissue sectioning with the C...

40m

6 Turn on the water bath and ensure that the temperature is between 34-37 °C

7 Prepare 1X PBS for use as a solvent for the embedding solution and as a buffer during tissue sectioning.

8 Place the chilling block (Figure 1a) into the -20 °C freezer for approximately 00:30:00 pri[illegible] to use.

30m

9 Prepare embedding solution in 40 mL of 1X PBS and heat in a microwave to dissolve. When the agarose and gelatin have dissolved, place in the preheated water bath between 34-37 °C to cool the solution down before embedding.

10 Cut the tissue into 10 mm^2 sections using the scalpel and place onto a petri dish. Cover the tissue in 1X PBS and place it On ice for approximately 00:10:00 (Figure 3c). Once the tissue is ready to be embedded, gently dab the tissue with blotting paper to remove excess moisture (6). 10m

Note

Agarose and gelatin gel blocks may be required for soft mucosal tissue e.g. porcine colon, to help stabilise it as otherwise the tissue will collapse against the side of the specimen tube, making embedding difficult. In these cases, add 2 mL of the liquid agarose and gelatin embedding solution to a petri dish and place it on ice to cool (approximately 3 mm in thickness). Once cooled, cut the gel into 10 mm^2 sections (7). Apply a small amount of superglue to the 10 mm^2 gel block and carefully stick the outer layer of the tissue sections to this block i.e. area of interest should be facing upwards. Leave to dry for approximately 00:01:00 .

11 Apply a small amount of superglue to create a thin layer on the surface of the white plunger of the specimen tube (Figure 1c) and carefully adhere one side of the tissue to the base of the white plunger using a tweezers, Figure 3d. Take care to orientate the tissue so that the area of interest is sectioned appropriately. This secures the tissue in place before the embedding solution is added to the tissue. Allow to dry for approximately 00:01:00 . 1m

12 Once dry, slide the metal tube of the specimen tube (Figure 1d) over the white plunger so that it is level with the height of the tissue, Figure 3d. The tissue is now ready for embedding with the embedding solution.

13 Hold the combined specimen tube containing the tissue sample at a 45° angle and slowly pipette in the agarose and gelatin embedding solution until the specimen tube is full. Ensure that no air bubbles are in the solution, Figure 3e.

14 Remove the chilling block from the -20 °C freezer and slide over the specimen tube for approximately 00:01:00 until a hard gel is formed, Figure 3f. 1m

15 Place the specimen into the holder on the buffer tray and secure in place using the thumbscrew. Add the chilled 1X PBS to the buffer tray, Figure 3g.

16 Attach the blade holder to the vibrating head unit by sliding it onto the axial bar and secure in place

with an Allen wrench.

17 As per the manual guidelines of the Compresstome®, begin with bigger cutting thicknesses e.g. 1 mm and work in reducing increments to the required thickness in approximately three cuts.

18 To mount the tissue for microindentation, gently remove sections from the buffer tray with a paintbrush and place on a drop of liquid agarose and gelatin in a petri dish. The tissue is kept hydrated in 1X PBS at approximately 0-4 °C before indentation to mitigate tissue degradation, Figure 3h,i. The fresh tissue sections should undergo microindentation within 2 hours of receiving the tissue into the laboratory. Disassemble as per the manual and clean glue off the embedding block and blade mount with acetone.

19

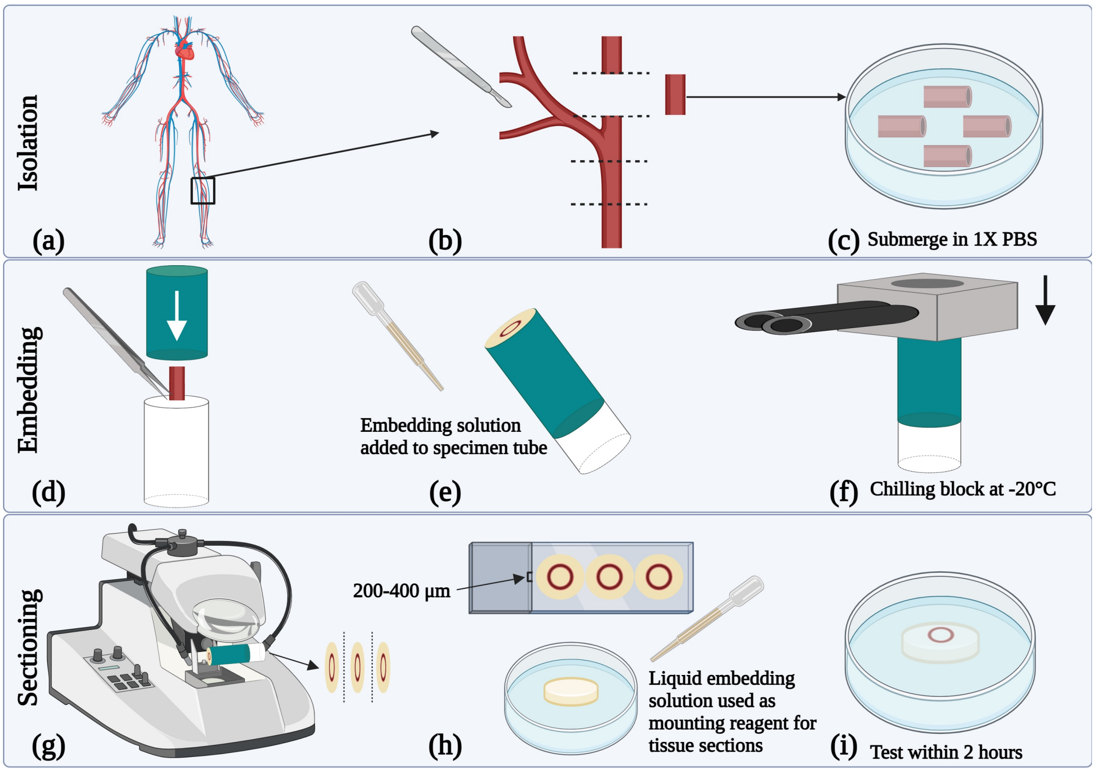

Figure 3 Example of tissue preparation, embedding and sectioning for microindentation using human vascular tissue. (a) Human tissue is obtained on the day of surgery. (b) A region of tissue is isolated and cut to approximately 10 mm^2 for microindentation. (c) Samples are hydrated in 1X PBS and placed on ice for approximately 10 minutes prior to embedding. (d) The tissue segment is glued to the white specimen tube and allowed to dry for 1 minute. The metal specimen tube is placed onto the white specimen tube once the glue is dry. (e) The agarose and gelatin embedding solution is added to the specimen tube. (f) The filled specimen tube is rapidly cooled with a chilling block or on ice for approximately 1 minute. (g) The cooled specimen tube is mounted into the vibratome. (h) The tissue is sectioned to the desired thickness. (i) The tissue section is mounted onto the liquid agarose and gelatin embedding solution. The tissue is hydrated in 1X PBS before microindentation (Created with BioRender.com).

Microindentation

1h

20 Microindentation is performed using the Chiaro Nanoindenter (Optics 11, the Netherlands), Figure 2b. Before each experiment, the probe is calibrated by submerging the cantilever in 1X PBS and using a glass calibration dish. The study is performed at room temperature to prevent measurement error due to temperature drift. The Petri dishes containing the tissue sample are mounted onto an Olympus IX73 microscope, thus allowing visualisation of the tissue during the experiment under the 10X brightfield setting. 1X PBS is added, *via* pipetting, to keep the tissue samples hydrated during the indenting process. Areas of damaged or torn tissue are avoided and indenting is performed away from the edge of the tissue. The probes used to perform this microindentation are within the 0.25 N/m cantilever stiffness and 50 µm radius spherical tip range of probes. An automated 3x3 matrix scan, spaced in 100 µm increments to avoid overlapping, is performed (3). Ultimately, this process allows for the suitable microindentation of fresh soft biological tissue.
