# Supplementary figures and images for: Microindentation of fresh soft biological tissue: A rapid tissue sectioning and mounting protocol
Source: PLoS One. 2024 Feb 29;19(2):e0297618. doi: 10.1371/journal.pone.0297618 (PMC10903917; doi:10.1371/journal.pone.0297618)

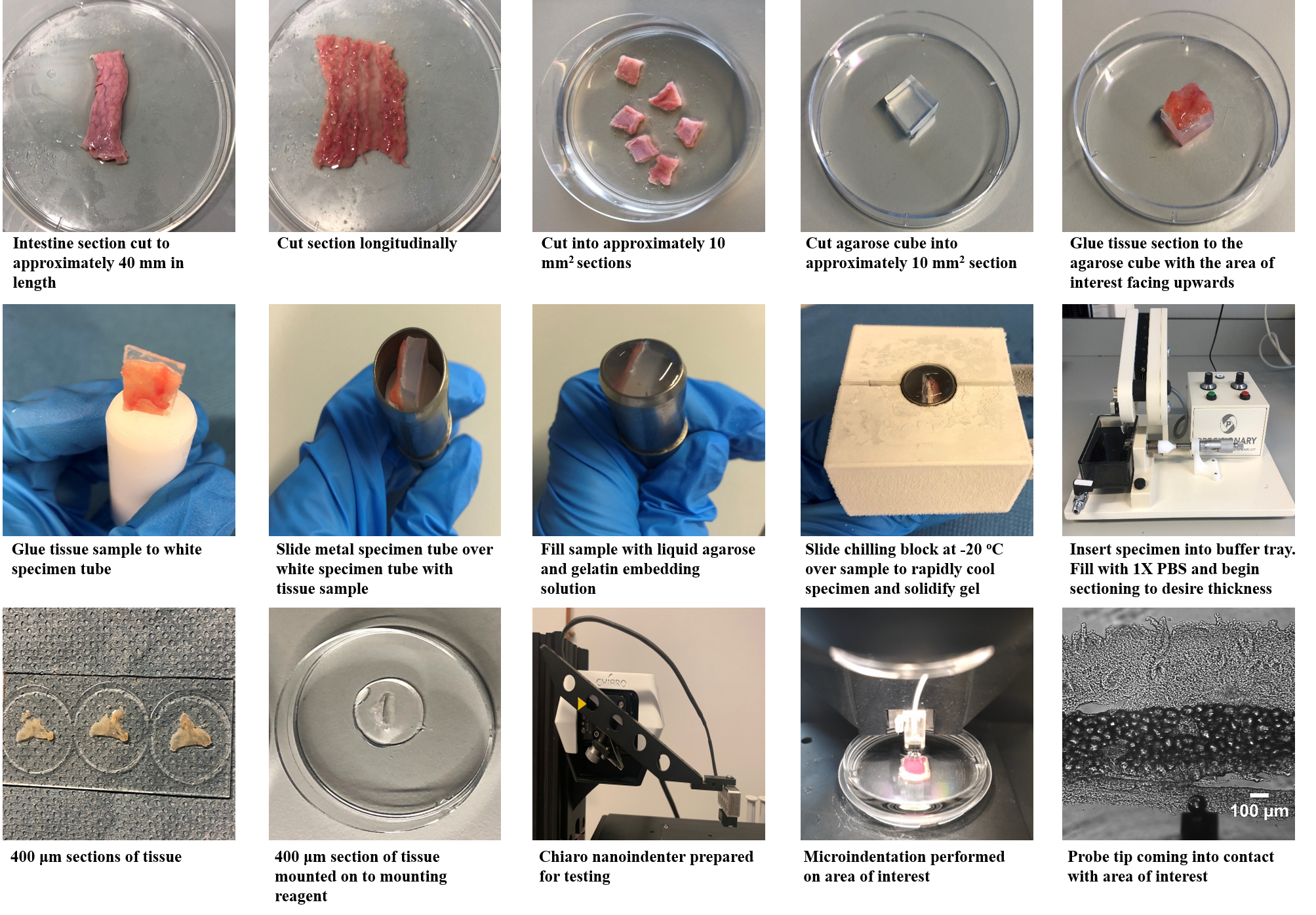

Supplement: S1 Fig — (TIF) [file pone.0297618.s003.tif]

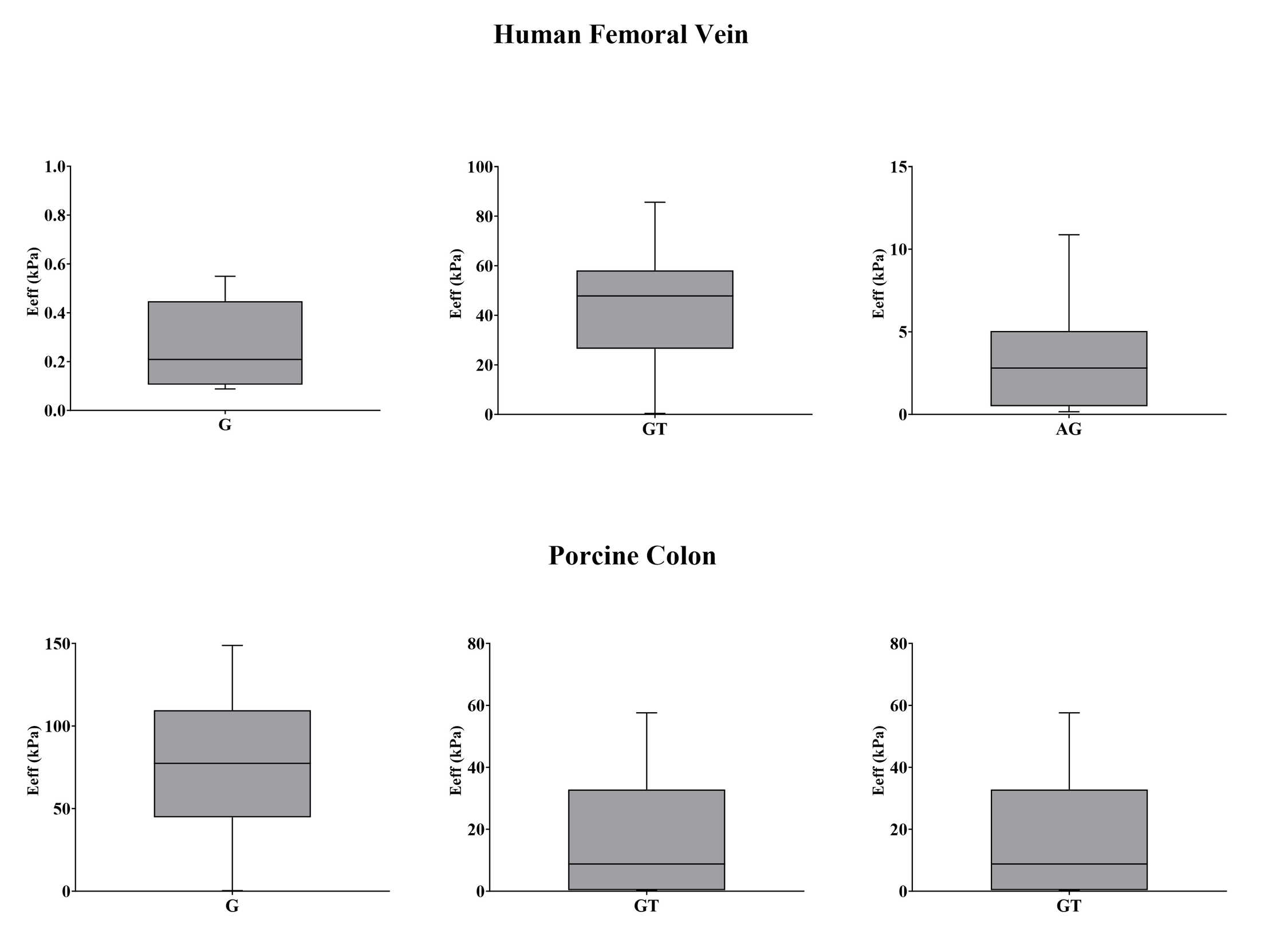

Supplement: S2 Fig — (TIF) [file pone.0297618.s004.tif]
